# A Public Health Perspective of Post-Traumatic Stress Disorder

**DOI:** 10.3390/ijerph19116474

**Published:** 2022-05-26

**Authors:** Ghazi I. Al Jowf, Ziyad T. Ahmed, Ning An, Rick A. Reijnders, Elena Ambrosino, Bart P. F. Rutten, Laurence de Nijs, Lars M. T. Eijssen

**Affiliations:** 1 Department of Psychiatry and Neuropsychology, School for Mental Health and Neuroscience (MHeNs), Faculty of Health, Medicine and Life Sciences, Maastricht University Medical Centre, 6200 MD Maastricht, The Netherlands; a.ning@maastrichtuniversity.nl (N.A.); ra.reijnders@maastrichtuniversity.nl (R.A.R.); b.rutten@maastrichtuniversity.nl (B.P.F.R.); laurence.denijs@maastrichtuniversity.nl (L.d.N.); 2 Department of Public Health, College of Applied Medical Sciences, King Faisal University, Al-Ahsa 31982, Saudi Arabia; 3 European Graduate School of Neuroscience, Maastricht University, 6200 MD Maastricht, The Netherlands; 4 College of Medicine, Sulaiman Al Rajhi University, Al-Bukairyah 52726, Saudi Arabia; 171110040@srcolleges.org; 5 Institute for Public Health Genomics, Department of Genetics and Cell Biology, Research School GROW (School for Oncology and Reproduction), Faculty of Health, Medicine and Life Sciences, Maastricht University, 6200 MD Maastricht, The Netherlands; e.ambrosino@maastrichtuniversity.nl; 6 Department of Bioinformatics—BiGCaT, School of Nutrition and Translational Research in Metabolism (NUTRIM), Faculty of Health, Medicine and Life Sciences, Maastricht University, 6200 MD Maastricht, The Netherlands

**Keywords:** stress, traumatic stress, PTSD, prevention, public health, treatment, biomarkers, burden

## Abstract

Trauma exposure is one of the most important and prevalent risk factors for mental and physical ill-health. Prolonged or excessive stress exposure increases the risk of a wide variety of mental and physical symptoms, resulting in a condition known as post-traumatic stress disorder (PTSD). The diagnosis might be challenging due to the complex pathophysiology and co-existence with other mental disorders. The prime factor for PTSD development is exposure to a stressor, which variably, along with peritraumatic conditions, affects disease progression and severity. Additionally, many factors are thought to influence the response to the stressor, and hence reshape the natural history and course of the disease. With sufficient knowledge about the disease, preventive and intervenient methods can be implemented to improve the quality of life of the patients and to limit both the medical and economic burden of the disease. This literature review provides a highlight of up-to-date literature on traumatic stress, with a focus on causes or triggers of stress, factors that influence response to stress, disease burden, and the application of the social-ecological public health model of disease prevention. In addition, it addresses therapeutic aspects, ethnic differences in traumatic stress, and future perspectives, including potential biomarkers.

## 1. Introduction

According to the Diagnostic and Statistical Manual (DSM) and the International Classification of Diseases (ICD), a traumatic experience is an event that can pierce into the integrity of an individual or a group causing distress, feeling of helplessness, horror, or intense fear reaction [1].

As reported by Terr (1991), trauma is categorized into two levels/domains, type 1 trauma and type 2 trauma. Type 1 trauma usually originates in childhood following unanticipated single events, typical in inducing post-traumatic stress disorder (PTSD). On the other hand, type 2 trauma follows repeated exposure to long-standing external events [2]. Importantly, type 2 trauma can also lead to the development of PTSD and other trauma-related reactions. It is worth mentioning that, while as much as 90% of the general population experience traumatic stress, only 20–30% of them develop PTSD [3].

The lifetime prevalence of traumatic stress ranges from 0.56% to 6.67% in Europe, with high prevalence rates in the Netherlands, the UK, France, and Germany [4]. Exposure to traumatic events is recognized as the essential key to developing stress-related disorders [5]. The most frequent disorder resulting from traumatic stress is PTSD [6]. In general, PTSD is a severe, chronic, and disabling disorder, which develops after exposure to a traumatic event in susceptible individuals, involving actual or threatened injury to themselves or others [7,8]. The most common stressor associated with PTSD is usually war and combat and witnessing, while in women, it appears to be sexual assault and rape [9]. Many models are developed aiming to predict the development of PTSD. A common aspect between these models is the interaction between the stressor, the peritraumatic condition, and the person’s susceptibility [10,11,12].

In the DSM-1, PTSD was not categorized as it is today; rather, it was categorized as a gross stress reaction, one of the transient personality reactions (along with adult situational reaction, adjustment reaction of infancy, adjustment reaction of childhood, adjustment reaction of adolescents, and adjustment reaction of late-life). Subsequently in the DSM-3 (introduced in 1980), PTSD was then recognized (for the first time as post-traumatic stress disorder) as “war neurosis” or “shell shock” as it was commonly seen during times of war. As the requirement for a person’s subjective negative response (defined as psychological distress or physiological reactivity to trauma-related cues) was eliminated as a diagnostic criterion, PTSD is no longer considered as fear- and anxiety-based disorder in the DSM-5 (introduced in 2013). Rather, it is categorized as a disorder arising in response to traumatic or stressful events preceding the emergence of its symptoms. According to the Diagnostic and Statistical Manual of Mental Disorders (DSM-5), PTSD has four components: intrusion symptoms, avoidance, alterations in cognitive function and mood, and arousal that impairs the individual’s functioning [1].

There are two sets of diagnostic criteria in the DSM-5 for PTSD, one for those of six years of age and older, and the other for children under six years of age. This discrimination was based on evidence that children below six years of age have a lower diagnostic threshold [13,14].

Differences in diagnostic definition and criteria of PTSD might hinder a precise and equal detection and diagnosis and can thereby limit effective and timely management [15]. In addition, it has been reported that declarative and short-term memory deficits may be susceptibility factors for PTSD [16]. Moreover, and as mentioned earlier, comorbidities (e.g., major depression disorder, anxiety disorders, and substance use disorders) further complicate the diagnosis and management of these comorbidities, demonstrating the need for the development of reliable diagnostic markers, such as biomarkers [17,18,19,20,21].

As part of management and patient care, prevention plays an important role in averting the disease before it develops or minimizing complications and morbidity if the disease already ensued. One way of doing so is by the implementation of prevention models, through which different levels of prevention (primary, secondary, and tertiary) can be enforced. Although out of the scope of this paper, it is worth mentioning that genetics and epigenetics have an important role in the interindividual differences in response to traumatic stress [22,23].

This review provides an insight into the burden of traumatic stress and its complications, some of the known causes of traumatic stress, factors believed to influence the response to stress, and integrating with the public health prevention model applied for traumatic stress. A literature search was performed using keywords to finds papers in PubMed, EMBASE, and SCOPUS. The report of literature in this paper aims to provide a general perspective of public health that demonstrates the importance of the early recognition of traumatic stress.

## 2. The Burden of Stress-Related Mental Disorders and PTSD

As mentioned earlier, PTSD remains the most frequently encountered disorder as a result of traumatic stress. Due to the high lifetime prevalence and significant consequences, the burden of the disease, both on the patient and the community, is expected to be high. Besides the burden on the patient, an economic burden and medical burden also exist.

### 2.1. Economic Burden

Stress-related mental disorders often have their onset gradually and at an early age [24], and are expected to cost the world USD 16 trillion by 2030 [25,26]. The economic burden can be mainly categorized into direct healthcare costs, productivity loss, societal cost, and non-healthcare costs. The general cost of diseases is usually classified into three types: direct, indirect, and intangible cost [27]. Direct costs include healthcare costs (diagnosis, treatment, and rehabilitation) and non-healthcare costs related to consuming non-healthcare resources, such as transportation, household expenditures, relocating, property losses, and informal care [27]. Indirect cost is productivity losses due to morbidity and mortality, borne by the individual, family, society, or the employer [27,28]. While intangible cost is not monetary but relates to function loss, increased pain, and reduced life quality, it can be regarded as an indirect economic burden and cost of illness [29].

The cost of psychiatric contact and outpatient treatment is surprisingly higher when compared to drug treatment and rehabilitation services [30]. The annual mean direct costs of PTSD per individual were much lower in South-Eastern European countries (USD PPP (purchasing power parities) 198–7110) compared to UK, Germany, and Northern Ireland (USD PPP 2337–26,991), probably due to the difference in healthcare spending between these countries. As expected, these numbers are negatively influenced by the severity of symptoms [30].

In Northern Ireland, the total economic burden in patients with PTSD as another example was GBP 172.8 million in 2008 values, including GBP 33.0 million in direct cost and GBP 139.8 million in indirect cost [31]. In Germany, the overall economic burden costs for PTSD account are EUR 43,000 per person. Mental disorders occupy 59%, which is the largest portion, and 18% of this value is for PTSD, with at least twofold more costs than a control group in 2013 [32].

Nearly all previous researchers found that indirect cost weighs similar or more in magnitude in the overall economic burden than direct costs [31,33,34].

### 2.2. Medical Burden

The medical burden is the impact a disease has on a population, which can be measured by indicators such as morbidity, mortality, and cost. The medical burden includes health care burden, comorbidity, and substance abuse, which also needs further treatments [35]. The medical burden of illnesses can be quantitatively measured by a cumulative illness rating scale [36]. This scale is a tool prevalently used as a criterion to evaluate medical burden in older adults as well as veterans [37,38].

Co-occurrence of mental as well as the general medical disorders are among the most common and disabling combinations, with approximately 30% of individuals with comorbidity having both a mental and a physical disorder [39]. In addition, 68% of adults with mental disorders have physical medical conditions [40]. Patients with medical comorbidity are in greater need of medical services with the loading healthcare system. For example, PTSD is exacerbated by comorbid medical illness, accounting for cumulative service utilization.

Returning veterans with PTSD, as an example, have a higher medical burden than those without mental health conditions [41]. In addition, these medical burdens are conditional on gender. Women tend to have more medical burdens than men. The median number of medical conditions for women with PTSD was seven, while for men was five [41].

Substance abuse disorders and social disadvantages can contribute to comorbidity and exacerbate its effect [39]. For example, co-occurring substance use and mental disorders are common among adults with opioid use disorder [42]. Thus, the soaring medical burden is associated with treatment resistance, medical comorbidity, and related substance abuse.

## 3. Types of Stressors

A stressor, or a stressful event, is the prime causative factor of PTSD, and is one of the criteria for diagnosing PTSD [43,44]. There are numerous types of stressors that may give rise to PTSD, including sexual assault, war and combat, child abuse and neglect, medical illnesses and disasters, in addition to others (Table 1).

### 3.1. Sexual Assault

Sexual assault is defined as “any form of sexual contact without voluntary consent, and that violates a person’s sense of autonomy, control and mastery over their body” [45]. The prevalence of PTSD due to sexual assault is 50%, making it the most common trauma resulting in PTSD in women [46]. Among those, rape is the most common form of sexual assault, but other forms also contribute to the trauma [46]. The dilemma of sexual assault is that it is a personal, individualized challenge to overcome and has a societal aspect that complicates the recovery process [46,47].

### 3.2. War and Combat

PTSD in military personnel is a common subject of psychiatric and psychological research, and tends to be correlated to the severity of the injury experienced [48]. Hoge et al. surveyed PTSD in US soldiers returning from Iraq months after returning from deployment. Their study showed that the prevalence of PTSD increased over the months, but interestingly, the severity of the physical injury was correlated to the earlier development of disease [49]. Additionally, some factors can affect disease development and progression. Of importance is deployment with combat experience, childhood adversity (adversity relating to family relationships and childhood antisocial behavior), leaving service, and serious accidents, as demonstrated by a study on British military personnel [50]. Important to note is that the effect of war is not only on soldiers that are deployed to the battlefield; the civilians are affected too. A systematic review on mental health outcomes among populations exposed to mass conflict and displacement found an overall prevalence of PTSD of 30.6% across all included studies (15.7% in studies > 1000 participants only). The main contributing factors retrieved were reported torture, cumulative exposure to potentially traumatic events, time since conflict, and assessed level of political terror [51]. Children are also strongly affected, not only by war trauma, but also by the effects this has on socio-ecological factors at family (parenting) and community levels [52]. This can also be seen in the 2022 Ukraine invasion, where multiple traumatic exposures have a critical impact on mental health [53,54].

### 3.3. Child Abuse and Neglect

In a prospective cohort study, children were followed up until their young adulthood, and both physical and sexual abuse increased the risk of developing PTSD. Another significant finding was that childhood neglect similarly increased the risk, and these factors independently contributed to the risk of the disease [55]. In 2009, Koenen et al. investigated whether there is a sex difference regarding these risk factors. They found a twofold difference in the risk of developing PTSD after childhood trauma, as women were found to have the highest increased risk among all the risk groups [56]. Additionally, those who experienced childhood trauma showed greater somatic symptoms, affect dysregulation, and suicidal behavior as compared with those without PTSD [57].

### 3.4. Medical Illness

It is becoming more evident than before that severe medical illnesses contribute to the risk of developing PTSD. Studies have been conducted on individuals with a specific disease to assess the degree of this risk. In a study on patients with Acute Coronary Syndrome (ACS), the prevalence rate of PTSD among 24 individuals was 12%, giving it the name ACS-induced PTSD [58]. Furthermore, patients with symptoms of PTSD that presented to the emergency room (ER) were more likely to be more worried about future stroke and have worries about medications, while not being adherent to medications [59]. Lastly, patients who experienced an intensive care unit stay showed a 24% PTSD prevalence 1–6 months after, as demonstrated in a meta-analysis [60].

### 3.5. Disasters

Disasters are traumatic events experienced by individuals that commonly lead to physical and mental consequences [61]. There are different types of disasters: natural disasters, such as tornados, floods, and hurricanes, and human-made/technology disasters, such as the Chernobyl disaster, terrorism, and torture [62]. The prevalence of PTSD was found to be higher in studies that focused on individual victims of disasters than on the general population [63]. Being female is considered to be a risk factor for the initiation of PTSD after disasters [64]. Other factors that found to be correlated with the initiation of PTSD after disasters included weak coping skills, external locus of control, history of previous trauma, low social support, media exposure, and others [65,66,67,68]. Furthermore, the degree of individual exposure to disaster is associated with PTSD likelihood [65]. The prevalence of PTSD is higher among the individuals who were directly exposed to the disaster and lower among individuals who rescued disaster victims [62].

**Table 1:** Main types of stressors related to PTSD.

Stressor Related to PTSD	Study	Findings
Sexual assault	Creamer et al. (2001) [46]	Most common traumatic stressor resulting in PTSD, accounting for 50% of cases; among these, rape was the most common form of sexual assault
War and combat	Hoge et al. (2008) [49]	Severity of physical injury is correlated to the earlier development of PTSD in soldiers returning from deployment
Child abuse and neglect	Koenen, Widom (2009) [56]	Childhood physical and sexual abuse, as well as neglect significantly increase the risk of developing PTSD. Females tend to have an increased risk
Medical illness	Edmondson et al. (2012) [58]	The rate of PTSD among ACS patients was 12%, while it was 24% for those who stayed in the ICU. Additionally, PTSD patients were more likely to not adhere to their medications
Disasters	Ahern et al. (2002) [68]	Female gender, low social support, history of previous trauma, and direct exposure to the disaster were all factors that correlated with PTSD initiation after the disaster

### 3.6. Other Factors

While the causes mentioned above represent the main and most important ones, it must be acknowledged that the causes of traumatic stress are diverse. Furthermore, it is worth mentioning that some factors are considered risk factors, and they include a low social-economic status, female gender, family history of mental illness, and prior mental disorders [69]. The COVID-19 pandemic deserves a mention here, as some authors attempted the assessment of its impact on mental health. A meta-analysis was conducted on the prevalence of post-traumatic stress symptoms and psychological stress with a pooled prevalence of 23.88% and 24.84%, respectively [70]. Bridgland et al. found that participants in their study had PTSD symptoms when directly (COVID-19 diagnosed) or indirectly (e.g., media coverage and lockdown) exposed [71]. Surprisingly, patients had symptoms in response to anticipated events, giving a new view that traumatic stress could be to anticipated future rather than only impact of past events [71]. Additionally, the sequential exposure to multiple stressors, such as the Ukrainian war trauma with the stress from the global pandemic of COVID-19 not completely over, can have disastrous effects [53].

## 4. Factors Moderating the Impact of the Stressor

### 4.1. Emotional Care

**Figure 1:**
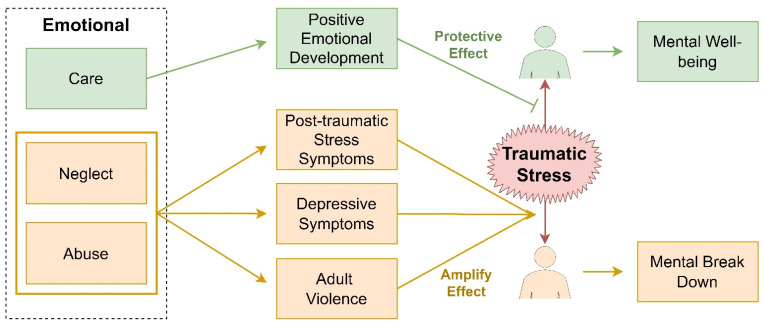
The impact of emotional care, emotional neglect, or abuse in early life on mental health in response to traumatic stress later in life.

Children during infancy should receive consistent and constant emotional care because it is a critical period of emotional, social, and cognitive development. The opposite of emotional care can be classified into two subtypes: one is active emotional abuse, which receives much attention, and the other is passive emotional neglect [72]. If emotional neglect occurred early in life, social and emotional rehabilitation deficiencies could be seen after being inflicted with traumatic stress [73,74,75]. A four-year longitudinal research on adopted children who experienced emotional neglect showed that children were in the clinical or borderline ranges for symptoms of post-traumatic stress arousal (19%), avoidance (14%), and intrusion (8%) [76]. Figure 1 provides a general overview of the information about emotional care discussed in this section.

Of all types of abuse, including childhood emotional abuse, physical abuse, and sexual abuse, only emotional abuse was independently associated with depressive symptoms, emotion dysregulation, and interpersonal problems in a cross-sectional study of 276 female college students [77]. In contrast, an emotionally responsive environment is found to possibly protect from severe traumatic stress even in those with genetic vulnerabilities [73,78].

Trauma-experienced youths tend to have emotional problems. A systematic review indicated that traumatized youths showed emotional regulation difficulties, including affect dysregulation, mood swings, affective and mood instability, or lability [79].

### 4.2. Age

An earlier age of trauma exposure is associated with an increased risk of PTSD development [73,80]. Within PTSD, youth age (9–17) is positively associated with the volumes of brain structures (amygdala), but this is not observed in the non-PTSD youth controls [81]. Thus, severe stress may influence age-related variation in brain structures. Furthermore, a study on combat veterans showed age-accelerated shrinking of the cortical surface area in some regions when combat-related mild traumatic brain injury and PTSD are present, a pattern that was not consistently found in those with mild traumatic brain injury only [82].

### 4.3. Number and Impact of Traumatic Events

Besides age, other modifiable risk factors, such as earlier traumatic events, have been associated with increased perceived severity of current traumas [69,73,83,84]. An investigation on 444 refugees from the 1994 Rwandan genocide showed that higher numbers of different lifetime traumatic event types were associated with a higher probability of lifetime PTSD [85]. One additional traumatic event experienced was associated with a 19% increase in the probability of developing lifetime PTSD [85]. This increase indicated an accumulative effect of traumatic events on the onset of PTSD. Besides, 314 college students were asked to rate the importance of different events, including interpersonal or non-interpersonal ones. The results show that perceived importance was higher for interpersonal than non-interpersonal events [86]. Therefore, both the traumatic event number and event type impact mental health in response to stress.

### 4.4. Education

Lower levels of education render subjects at higher risk of PTSD in a study investigating emergency health care personnel in Italy [87]. Another study showed that a lower education level with other factors, such as race and age of combat exposure, predicts the current PTSD symptoms and symptom exacerbation in the longitudinal study on Vietnam veterans 40 years after the combat [88]. People with lower education levels have higher scores on the Kessler 10 scale, indicating more anxiety and depression than people with higher education in a cross-sectional study that included people who lived in Syria in different governorates [89].

### 4.5. Gender

The lifetime prevalence of PTSD is different between genders with higher rates among females (10–12% vs. 5–6% among males). Explanations for this are both psychosocial (e.g., type of trauma, as women are exposed to high-impact trauma, e.g., sexual assault, as described earlier) and biological (e.g., lower oxytocin release, a hormone that has been shown to reduce PTSD development as discussed later) [90]. Women veterans reported the highest lifetime and past-year PTSD rates compared with women civilians, men veterans, and men civilians [91]. However, another study on US military personnel deployed in support of the operations in Iraq and Afghanistan showed no significant gender differences for the likelihood of developing PTSD or for PTSD severity scores [92].

### 4.6. Race

Race has been reported as an impact factor mediating traumatic stress (race-based traumatic stress), largely due to race discrimination rather than biological reasons [93]. A study on 421 community-based adult respondents showed that race-based traumatic stress is significantly related to trauma symptoms, especially in people who consider negative race-based experiences stressful [93]. Empirical data in 2012–2017 suggests that, in the US, Latino Americans, African Americans, and Native Americans tend to present with the highest rates of PTSD, while Asian Americans tend to present with the lowest [94].

To our knowledge, African Americans have the highest prevalence rate of PTSD across all ethnicities [95,96,97]. Although not fully understood, this disparity between ethnicities might arise from a difference in traumatic exposure or the pre-exposure vulnerability [96,98,99]. Research has been conducted in this regard, but several factors are implicated in this difference. For example, higher PTSD among African Americans might be due to racism and verbal assault, stigmatization, and the discrimination perceived by themselves [100,101,102].

On the other hand, some factors might account for the lower PTSD prevalence among other ethnicities, such as better socioeconomic status, higher education, and higher income [69,103]. Additionally, other psychiatric disorders, such as depression and anxiety, were associated with a higher risk of developing PTSD, and although these disorders are more prevalent in other populations, the risk of developing PTSD is found to be higher in African Americans [104,105]. It also appears that sociopathy alcohol and drug abuse, which are seen to be lower in Asians, could contribute to the ethnic difference and might explain the lower PTSD prevalence in that group [106].

In 2017, Alexander and his colleagues investigated the ethnic difference in PTSD vulnerability following hurricane Katrina [107]. The two-fold higher odds of African Americans compared to other populations to screen positive for PTSD was related to some factors, including worse prior mental distress, more stressful events, and less social support. Between these factors, only pre-hurricane mental distress has been shown to reduce this ethnic disparity. This might mean that hurricanes trigger the manifestation of PTSD of delayed onset, presumptively [107]. Overall, the retrospective nature of these studies might be seen as a limitation to the investigation of this ethnic association, and future prospective studies need to investigate the differences and arrive at a more solid conclusion.

Besides the likelihood of race discrimination exposure, ethnic origin can be a risk factor of traumatic stress mediated by culture. A study including Shanghai and Hong Kong residents and Americans showed the cultural differences in dialectical thinking, self-construal, and familyism in mediating resilience capacity. Dialectical thinking is the cognitive tendency toward attempts to reconcile two opposing perspectives or acceptance of contradiction [108]. Self-construal can be the independent self-construal common in the West or the interdependent self-construal common in East Asian countries. Similarly, tendency towards familyism (which means interests and gains are conceived at the level of the familial group rather than at the individual level) is present more in East Asian countries [94]. The study on three regions showed that independent self-construal, familyism, and dialectical thinking significantly mediated the relationship between culture and resilience capacity [108].

## 5. Public Health Model for Prevention and Intervention

### 5.1. The Social-Ecological Model

**Figure 2:**
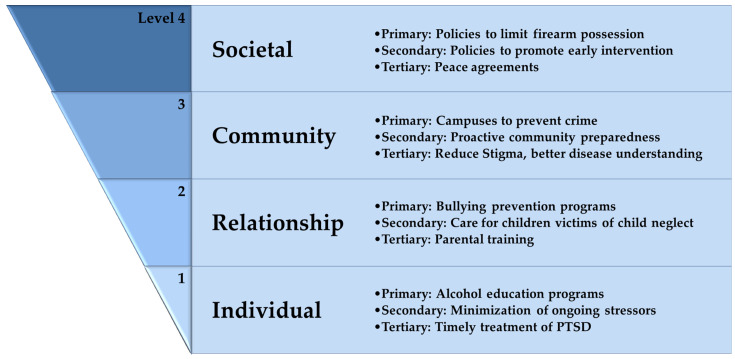
The social-ecological model of disease prevention as applied to PTSD. For each of the four levels, examples of primary, secondary, and tertiary prevention are given.

From a public health perspective, approaching a disease starts by identifying the causes and triggers, after which prevention (rather than treatment) can be applied at different stages of the disease development, with the aim of decreasing the disease burden at all levels. One model is the social-ecological model, a four-level model that aims to identify the factors contributing to disease development and poor health outcome at these levels: individual, relationship, community, and society [109,110] (Figure 2).

At the core of the model, the individual level is found, which includes personal characteristics, including biological and others (e.g., genetics, comorbidities, education level, and economic status). The next level, which is the relationship, includes one’s close social connections that exert influence over the individual (parents, partners, family, close friends, etc.). The third level is the community, which explores one’s contact with one’s community, which happens at a wider social level (e.g., schools, universities, meeting places, and workplace). The final level, the societal level, looks at how the society in which the individual lives can affect his health outcome, and is usually a cultural and political level (e.g., cultural habits, norms, societal education, economy, and policies). At each one of these levels, prevention is feasible, and if suitable, intervention can also be applicable [109].

The prevention applied at each level can be primary, secondary, or tertiary. Primary prevention focuses on recognizing individuals at risk and preventing disease development in a disease-free individual. Secondary prevention aims at intervening early after disease occurrence, to achieve cure if possible, or to control disease progression. Tertiary prevention aims at reducing disabilities resulting from the disease to maintain a better quality of life [111]. While it is easier to define a clear onset in medical (or physical) diseases, disorders resulting from traumatic stress are diagnosed based on specific criteria, which are sets of symptoms with duration. This might present a challenge to differentiate between primary and secondary prevention, as asymptomatic disease might be the case in many individuals [43].

### 5.2. Examples of Preventive Measures at the Multilevel Social-Ecological Model

As primary prevention aims at preventing disease occurrence, in a trauma situation, it aims at preventing exposure to trauma. As discussed earlier, there are many causes of trauma, and specific interventions can be directed at these causes. At the individual level, interventions can be in the form of educational programs on the risk of alcohol drinking and firearm acquisition for the youth and parental guidance for young children, as well as college programs to educate young adults about traumatic experiences [112]. Additionally, psychoeducation programs target military personnel prior to deployment on trauma reaction [113]. At the relationship level, parental and caregiver guidance and education can aim at reducing traumatic experiences for the children, for example, bullying at school or programs to reduce assaultive violence. At the community level, interventions include community support services, neighborhoods, and streets surveillance campuses. At the societal level, policies can target reducing the acquisition of firearms and alcohol consumption, as well as recalling defective motor vehicles and street maintenance [114] (Figure 2).

Secondary prevention is implemented after exposure to trauma and disease development, but the key is early intervention to prevent disease progression. At the individual level, and for trauma-exposed individuals, preventing ongoing exposure to stressors can halt disease progression [115]. Additionally, building prediction tools based on the susceptibility to develop PTSD after trauma exposure can identify high-risk patients, and hence, provide opportunities for intervention [43]. In addition, early psychological interventions can be effective [116]. At the relationship level, caring for relatives of domestic violence or children who are victims of child neglect can be useful. Medical intervention (e.g., cortisol and adrenergic medication administration) immediately after trauma exposure can take part in the secondary intervention, but weighing the harms and benefits should be determined carefully [117]. At the community level, providing shelters in the aftermath of disasters and making rehabilitation programs are strategies taken. Not only these, but also preparedness and measures in anticipation of disasters and traumatic experiences can be considered secondary prevention [118]. At the societal level, policies addressing early medical intervention, as well as screening campuses can limit disease progression (Figure 2).

When interventions are aimed at preventing the progression of disease and development of disabilities, they are considered part of the standard care and treatment of the disease. At the individual level in tertiary prevention, seeking medical care and compliance with treatment, as well as better knowledge of the disease and complications helps to prevent disabilities. At the relationship level, special training for care from parents, relatives, and friends to patients undergoing therapy can be helpful. As demonstrated by Leve and colleagues, foster parents training for the care of trauma-exposed children with psychological illness halts disease progression [119]. Both community and societal levels fall under political influence, and measurements to promote community understanding of the disease, reducing stigma, and ensuring peace and fighting violence all play an important role in preventing disability [118,119,120] (Figure 2).

### 5.3. Secondary Prevention and Treatment and Modalities

While the absolute avoidance of traumatic stress is often not a matter of control, the prevention of its complications can be pursued. After exposure to traumatic stress, individuals become vulnerable to complications, with PTSD being the most common. Several interventions are currently either implemented or under research to prevent such complications. These include behavioral therapy (e.g., cognitive behavioral therapy (CBT), prolonged exposure (PE), and eye movement desensitization and reprocessing (EMDR)) as well as pharmacological agents.

Being an effective and important therapeutic modality, CBT aims to give patients a sense of control over their fears, using different methods, such as exposure, to achieve fear extinction and support cognition to change the patient’s perception about the trauma [121]. Several clinical studies have been conducted on the effect of CBT early after trauma exposure to reduce complications and the appearance of symptoms. In 2021, Rothbaum and her colleagues investigated the effect of prolonged exposure CBT on patients who experienced traumatic stress, such as rape and motor vehicle accidents. It appeared that exposure-based CBT reduced PTSD symptoms at the assessment, especially for victims of sexual assault [122]. In a population of cardioverter defibrillator patients, CBT in less than two months for eight weeks significantly lowered the development of symptoms and promoted patient improvement in the CBT group [123]. Besides CBT, other therapies, such as PE in which the patient processes the traumatic event, as to decrease distress at further recall [124], and EMDR in which eye movements can reduce the intensity of traumatic memories [125], were the most utilized therapies in evidence [126,127]. The new consolidation/reconsolidation therapy is based on memory processing and the modulation of this process by pharmacologically interfering with this process during the recall of the disturbing memory, and to date, it has shown promise [128]. In this systematic review, Forneris et al. assessed the efficacy and comparative effectiveness of different psychological and pharmacological interventions in reducing the incidence or severity of PTSD symptoms. Interventions such as CBT reduced symptoms severity, while other interventions, such as debriefing, did not show evident benefit. The evidence for several other approaches appears to be limited and insufficient due to shortcomings in many included studies [129].

The other intervention modality is the pharmacological one, and many drugs have been tested to prevent complications. In addition, the findings from meta-syntheses showed that selective serotonin reuptake inhibitors (paroxetine and sertraline) and noradrenaline were effective and thus are highly recommended in the current guidelines [1,130].

Interestingly, propranolol helps to reduce traumatic recall in patients, given that it is administered early within hours after the traumatic exposure to affect memory formation and reduce traumatic recall [131]. A study on 64 trauma patients tested the effect of hydrocortisone compared to a placebo group. The symptom severity in the hydrocortisone group was lower than the placebo group, as the patients reported lower scores on the clinician-administered PTSD Scale (CAPS) [132].

The effects of oxytocin in the prevention of PTSD seem complex. A single administration had adverse effects on symptoms as it increased fear processing, while repeated administration reduced the development of PTSD symptoms [133]. The recreational drug 3,4-methylenedioxymethamphetamine (MDMA), when combined with psychotherapy, was associated with improved symptoms and lower functional compromise greater than psychotherapy and placebo [134]. Although trials showed controversial results, the alpha-blocker agent prazosin is used in clinical practice, as it appears to reduce symptoms [135].

The clinical implementation of the interventions above is harder than it seems, and the choice of a single standard therapy modality might not be effective. For that, a tailored and collaborative strategy for patients at risk is recommended. Zatzick et al. (2013) developed a randomized trial in which trauma survivors underwent stepped combined care management, psychopharmacology, and cognitive behavioral therapy compared to the usual care control condition. As expected, this therapy strategy reduced PTSD symptoms and improved functioning compared to the usual care group [136]. However, CBT is the mainstay of early prevention modality, but the challenging nature of implementation elicits the need to develop other modalities suitable for the needs of the particular subjects.

## 6. Future Perspectives: Biomedical Markers

Although PTSD is often a highly debilitating psychiatric disorder, no medical tools are currently available to prevent or minimize the impact of traumatic stress on mental health. PTSD symptoms prevent suffering individuals from leading a healthy lifestyle and are debilitating on a personal, societal as well as a professional level. Moreover, the economic burden of PTSD is substantial. There is thus a pressing need to develop additional tools to help PTSD prevention and treatment, such as biomarkers. We focus on biomarkers that aid the processes of diagnosis as well as determining therapy and response to treatment, supporting stratified precision medicine.

Integrating biomarkers along with the clinical assessment would provide a powerful means of managing PTSD and other psychiatric disorders. In addition to the role of biomarkers in the diagnosis and prediction of the onset of disorder, first (relative small scale) studies on biomarkers related to response to treatment are under investigation for possible future applicability [137]. For example, Felmingham et al. found reduced right amygdala activity and increased right anterior cingulate cortex activity in patients successfully treated with CBT [138]. Another example is the association between rostral anterior cingulate cortex (rACC) volume and the reduction in PTSD symptoms [139]. The same study demonstrated that activation of the ventral anterior cingulate and amygdala predict a better response to therapy [139]. On the side of pharmacotherapy, a promoter-region polymorphism, namely the LL 5HTTLPR genotype, was associated with a better responsiveness to sertraline (SSRI) [140]. Snijders et al. investigated the diagnostic potential of miRNA in a pilot study of patients with PTSD. In their pilot study, miR-138-5p was found to be significantly higher in PTSD patients as compared to controls. Additionally, only miR-1246 was significantly downregulated in PTSD cases compared to resilient subjects [141]. Although biomarkers showed promising initial results in predicting and diagnosing PTSD, further dedicated research is needed to determine the applicability of these biomarkers. Additionally, ethical considerations related to biomarkers for PTSD should be given attention: whereas prevention of avoidable harm and suffering can be considered a moral duty, the availability of such markers can also raise some concerns as to whether a test can be made obligatory and what the social and professional consequences of a susceptible or resilient status will be [142].

## 7. Conclusions

This paper provided a literature review of PTSD with the focus on traumatic stress prevention form a public health perspective. A traumatic experience is an event that can pierce into the integrity of an individual or a group causing distress, feelings of helplessness, horror, or intense fear reaction. The cause of this traumatic experience might range from war, terrorism, and disasters to sexual assault and child abuse. A well-recognized complication of such experience is PTSD. Current descriptive and empirical evidence showed race, gender, and age differences in the risk of developing PTSD, resulting in interindividual differences in disease manifestation. Early recognition and diagnosis help in the application of different levels of prevention (primary, secondary, and tertiary prevention), improving the course of the disease and limiting the complications, while reducing costs and burden of the disease.

Additionally, the implementation of preventive measures according to public health models of disease prevention can be seen as a means to achieve these goals effectively. A widely accepted model, the socio-ecological model, was implemented to study prevention at the levels of the individual, relationship, community, and society. While a variety of different prevention and treatment modalities exist for PTSD, including behavioral and pharmacological interventions, the identification of the suitable strategies is important to avoid treatment failure and relapses.

The earlier described differences in PTSD definition and lack of definitive diagnostic tools may be a contributing limitation to research on PTSD as well, which as such leads to some differences among studies and their conclusions and, thereby, our reporting thereof. A more detailed mapping of the definitions used across the several studies and possible inclusion of such criteria in a meta-analysis or systematic review could be a valuable future endeavor. The finding of biomarkers may help to synchronize diagnostic criteria, but also depends on the definition of disease classes for their discovery.

Although biomarkers showed promising initial results in predicting, diagnosing, and treating PTSD, further dedicated research is needed to replicate and validate these and (if successful) test for the clinical applicability of these biomarkers. Integrating biomarkers along with the clinical assessment may provide added value for diagnosing PTSD and prediction its course.

## Data Availability

Not applicable.
